# Anévrisme de l'artère mésentérique supérieure révélateur de la maladie de Behçet: à propos d'un cas

**DOI:** 10.11604/pamj.2015.20.312.5736

**Published:** 2015-03-31

**Authors:** Youssef Bibiche, Nabil Kanjaa

**Affiliations:** 1 Département d'Anesthésie Réanimation, Centre Hospitalier Universitaire Hassan II, Fès, Maroc

**Keywords:** Artère mésentérique supérieure, angioscanner, anévrisme, maladie de Behçet, superior mesenteric artery, CT angiography, aneurism, Behçet's disease

## Abstract

L'atteinte artérielle est une complication rare au cours de la maladie de Behçet. Elle représente un des modes d'expression de cette pathologie. Nous rapportons une observation d'un jeune homme de 25 ans qui était hospitalisé pour une douleur abdominale intense. L'angioscanner abdominaux a permis de poser le diagnostic d'un anévrisme d'une branche de l'artère mésentérique supérieure. Le patient a été opéré en urgence et les suites opératoires étaient simples. L'enquête étiologique de cet anévrisme a conclu à la maladie de Behçet. À la lumière de cette observation, nous insistons sur la nécessité de rechercher avec acharnement les symptômes de la maladie de Behçet devant une atteinte artérielle chez un sujet jeune. Le traitement de l'anévrisme doit être urgent.

## Introduction

La maladie de Behçet (MB) est une vascularite systémique fréquente dans les pays méditerranéens [1, 2]. L'atteinte vasculaire est une complication rare au cours de cette pathologie [1]. Elle représente un des modes d'expression de la maladie [1]. Nous rapportons un cas rare d'anévrisme d'une branche de l'artère mésentérique supérieure (AMS) révélant la MB et illustrant les difficultés du diagnostic étiologique.

## Patient et observation

**Figure 1:**
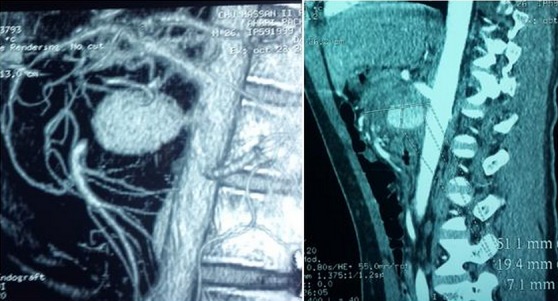
Reconstructions dans le plan sagittal montrent que l'anévrisme se développe aux dépens de l'artère mésentérique supérieure

**Figure 2:**
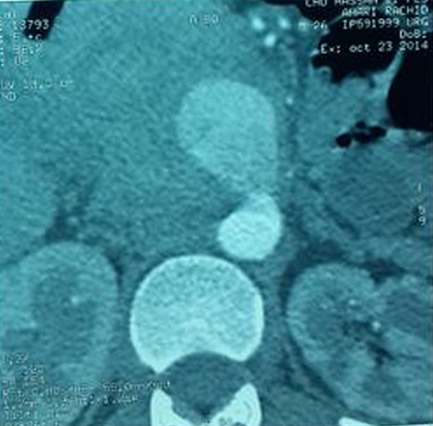
Angioscanner abdominal en coupe axiale montrant l'anévrisme

Un homme âgé de 25 ans consultait pour une douleur abdominale intense et isolée. L'examen clinique était sans particularité. L’échographie abdominale montrait une masse kystique pelvienne médiane de 4 cm de diamètre qui s'allumait au doppler couleur et présentait un flux de type artériel au doppler pulsé. Devant cet aspect, le diagnostic d'anévrisme a été posé mais l'origine n'a pu être déterminée. Un angioscanner abdominal a été réalisé. Il a confirmé le diagnostic d'anévrisme sacculaire développé aux dépens d'une branche de l'artère mésentérique supérieure (Figure 1 et Figure 2). Le malade était opéré en urgence et les constatations peropératoires étaient compatibles avec les données de l'imagerie. Les suites opératoires étaient simples. Le bilan étiologique trouvait à l'interrogatoire une aphtose buccale et à la biologie un syndrome inflammatoire avec un pathergy test positif. Devant l’âge du patient, l'aphtose buccale et le syndrome inflammatoire, le diagnostic de la MB a été retenu.

## Discussion

La maladie de Behçet a été rapportée pour la première fois par Hulusi Behçet en 1937 [1]. C'est une vascularite inflammatoire, multisystémique. Elle est caractérisée par une triade associant une aphtose buccale et génitale récidivante et une atteinte oculaire [2, 3]. La maladie de Behçet touche le sujet jeune, en principe de sexe masculin. L'atteinte semble plus fréquente dans les pays du moyen orient (2 à 44%) et méditerranéen (25,7%) [1, 3]. Notre patient est un homme âgé de 36 ans. Le diagnostic de la maladie est clinique et repose sur les critères de l'International Study Group for Behçet Disease [4]. Les atteintes les plus souvent rencontrées sont oculaires, cutanées, nerveuses, neurologiques et vasculaires. Au cours de cette pathologie, l'atteinte vasculaire peut être veineuse et/ ou artérielle. L'atteinte veineuse est plus fréquente que l'atteinte artérielle et intéresse tout le réseau veineux [1, 2].

Les manifestations artérielles sont en revanche beaucoup plus rares [1, 2, 5–7]. Le délai moyen de leur survenu est de 7,2 ans par rapport aux premiers signes avec des extrêmes allant de 2 à 20 ans [3]. Parfois, le diagnostic d'une maladie de Behçet est posé lors des complications vasculaires [2, 3]. Cela fait l'originalité de notre observation où l'anévrisme de l'artère mésentérique supérieure était le mode de révélation de la maladie de Behçet. Toutes les artères peuvent être touchées quel que soit leur calibre avec une prédominance pour l'aorte abdominale, les artères pulmonaires et fémorales [2, 8]. L'atteinte de l'artère mésentérique supérieure est rare (5,5% de tous les anévrismes viscéraux) [5, 9, 10]. L'anévrisme de l'artère mésentérique supérieure peut complique plusieurs pathologies: athérosclérose, infection, traumatisme, vascularites [9–11]. Le diagnostic de La maladie de Behçet doit être suspecté chez un jeune adulte qui présente un anévrisme artériel et les signes cliniques en faveur doivent être recherchés par un interrogatoire et un examen vigoureux, souvent itératif [2, 3]. L’échographie abdominale est le premier examen à demander devant une douleur abdominale intense. Couplée au doppler couleur et pulsé, elle permet de poser le diagnostic en montrant une augmentation du calibre de l'artère. Comme c’était le cas pour notre patient, parfois l'origine de l'anévrisme est difficile à préciser d'où l'intérêt de l'angioscanner. Ce dernier confirme le diagnostic, précise l'artère en cause grâce aux reconstructions multiplanaires et cherche d'autres atteintes non vues à l’échographie doppler. Il fournit un bilan lésionnel complet préthérapeutique.

Chez notre patient, l'angioscanner a permis de confirmer les données de l’échographie doppler et de préciser l'origine de l'anévrisme qui était aux dépens d'une branche de l'AMS. L'angio-IRM semble être particulièrement prometteuse et suffisante pour le bilan préthérapeutique [12]. L'angiographie n'a plus de place aujourd'hui dans le diagnostic positif mais elle occupe le premier temps d'un éventuel traitement endovasculaire de l'anévrisme. Devant le diagnostic d'un anévrisme de l'artère mésentérique supérieure, le traitement doit être urgent vu le haut risque de rupture [9, 11]. La chirurgie sur de tels terrains inflammatoires n'est pas toujours facile en raison des complications postopératoires dont la plus fréquente est la désunion anastomotique [10, 12] et tardivement les récidives qui peuvent engager le pronostic vital [3]. Actuellement le traitement endovasculaire est une nouvelle alternative du traitement chirurgical. Notre patient a été opéré en urgence et les suites opératoires étaient simples.

## Conclusion

La maladie de Behçet peut se présenter avec des manifestations artérielles. Le diagnostic doit être suspecté devant une atteinte artérielle chez un jeune adulte. L'imagerie en particulier l'angioscanner et l'angio-IRM permettent de confirmer le diagnostic et de fournir un bilan prèthérapeutique complet. Le traitement endovasculaire est une nouvelle alternative du traitement chirurgical dont les résultats peuvent être décevants en raison des complications postopératoires.

## References

[1] Benamour S, Zerouaz B, Benni R, Amraoui A, Bettal S (1990). Maladie de Behçet:316 cas. Presse Med..

[2] Filali-Ansary N, Tazi Mezalek Z, Mohattane A (1990). La maladie de Behçet, 162 observations. Ann Med Interne (Paris)..

[3] Amahzoune B, Boulahya A, Selkane C, Ait Houssa M, Bekkali Y, Arji M (2002). Manifestations artérielles de la maladie de Behçet: à propos de 5 cas opérés. Arch Mal Coeur Vaiss..

[4] (1990). International study group for Behçet's disease. criteria for diagnosis of Behçet disease. Lancet.

[5] Hamza M (1987). Large artery involvement in Behçet disease. J Rheumatol..

[6] Morimoto N, Okita Y, Tsuji Y, Inoue N, Yokoyama M (2003). Inferior mesenteric artery aneurysm in Behçet syndrome. J Vasc Surg..

[7] Kraiem S, Fennira S, Battikh K, Chehaibi N, Hmem M, Slimane ML (2004). Maladie de Behçet: cause rare d'infarctus du myocarde. Ann Cardiol Angeiol (Paris)..

[8] Basaran M, Sever K, Kafali E, Ugurlucan M, Alpagut U, Dayoglu E (2005). Les manifestations vasculaires de la maladie de Behçet: à propos d'un cas. Ann Cardiol Angeiol (Paris).

[9] Guinier D, Denue PO, Mathieu P, Landecy G, Heyd B, Mantion GA (2004). Giant superior mesenteric artery aneurysm. J Am Coll Surg..

[10] Dorigo W, Pulli R, Innocenti AA, Anichini C, Azas L, Barbanti E (2004). Isolated inflammatory aneurysm of superior mesenteric artery: unexpected pathologic diagnosis. J Vasc Surg..

[11] Jindal R, Natt N, Pandey V, Jenkins M (2005). A ruptured mycotic aneurysm of a branch of the superior mesenteric artery and pulmonary tuberculosis. Eur J Vasc Endovasc Surg..

[12] Bennouna-biaz F, Ourhouk M, Senouci K, Hassen B, Heid E, Lazrek B (1995). Maladie de Behçet, profil épidémiologique. Maghreb Med..

